# The effects of varying inertial loadings on power variables in the flywheel romanian deadlift exercise

**DOI:** 10.5114/biolsport.2022.106159

**Published:** 2021-07-03

**Authors:** Joey O Brien, Declan Browne, Des Earls, Clare Lodge

**Affiliations:** 1 HealthCore, Department of Science and Health, Institute of Technology Carlow, Carlow R93 V960, Ireland

**Keywords:** Iso-inertial device, Eccentric overload, Inertia load, Power output

## Abstract

The aim of this study was to investigate the effect of four different inertial loads (0.025, 0.050, 0.075, and 0.100 kg· m²) on concentric (CON) power, eccentric (ECC) power, and ECC overload in the flywheel Romanian deadlift (RDL). Fourteen recreationally trained males (27.9 ± 6.4 years, 90 ± 10.7 kg, 180.7 ± 5.5 cm) volunteered for the study. They had a minimum of two years of resistance training experience, although none had experience in flywheel inertia training (FIT). All participants performed the flywheel RDL on a flywheel device (kBox 3, Exxentric, AB TM, Bromma, Sweden). Each set was performed using different inertial loads, those being 0.025, 0.050, 0.075, and 0.100 kg·m². For CON, ECC power, and ECC overload, there was a significant difference (p < 0.001) between inertial loadings. In conclusion, results highlight that lower inertial load leads to higher peak CON and ECC power values, precisely 0.025 kg· m². Regarding ECC overload, medium to higher loads (0.050, 0.075, and 0.100 kg·m²) will lead to higher values.

## INTRODUCTION

Flywheel inertial training has recently become increasingly popular as a training modality. FIT was first researched over 20 years ago as a gravity-independent training method to counteract microgravity’s deleterious effect on skeletal muscle [1]. During FIT, subjects rotationally accelerate a flywheel during the CON muscle actions. The force applied unwinds a cord connected to the flywheel’s shaft, which starts to rotate and store energy [2]. Once the CON action is completed, the cord rewinds, and the athlete must resist the pull of the flywheel using an ECC muscle action to decelerate the wheel. By resisting the inertial force gently during the first third of the ECC action and then applying maximal effort to stop the movement at the end of the range of motion, brief moments of ECC overload can be produced [3]. ECC overload is when the ECC force is larger than the CON force during an exercise [4].

Previous research has revealed FIT potential for positive effects on maximal strength [5–7] maximal voluntary contractions [8–10], power output [5, 11], jump ability [6, 12], running velocity [13], ECC force production [14] and Post-activation performance [15]. Two previous meta-analyses [5, 16] reported FIT’s effectiveness in improving muscle hypertrophy, strength, power, and other performance characteristics. However, another meta-analysis [17] reported that FIT did not provide any additional benefits to muscle strength when compared with gravity-dependent resistance training.

There is a multitude of research investigating the effectiveness of FIT in improving strength and power output, but there are minimal practical guidelines regarding inertial loading.

An important variable that can affect FIT performance is inertial loading. Its importance during FIT lies in the fact that different inertial loads cause notable differences in the force-velocity curve [3], with the majority of preceding investigations, based on replication of previous research [18]. Significantly different values were reported in a previous study [19] in both power and ECC overload depending on inertial loading (six inertial loads ranging from 0.0125 to 0.100 kg·m²). In-relation to strength development, previous research has recommended using higher inertial loads (0.050–0.100 kg·m²) [4, 18, 20]. However, another study indicated no additional ECC overload stimulus with inertial loads beyond 0.0375 kg·m² [19]. Regarding power development and in contrast to strength development, recommendations suggest lower inertial loadings are advised to augment power output [4, 18, 21–23] but again, the recommended inertial loads vary greatly (0.0125–0.050 kg·m²).

Taking into account recommendations from previous studies regarding inertial loading and repetitions in strength and power development, the aim of this study was to investigate the effect of four differential inertial loads (0.025, 0.050, 0.075, and 0.100 kg· m²) on both CON and ECC power output and ECC overload in a flywheel RDL. Previous research has investigated the effect of inertial load on power output in both the flywheel squat [18] and flywheel leg curl [23] exercises, but to the best of our knowledge, no past study has investigated the effects of inertial loading in the flywheel RDL exercise. A recent study [24] investigated flywheel RDL effect on bicep femoris long head (BFlh) architecture, eccentric hamstring strength and sprint performance in Australian footballers. The study was conducted over a 39 week period that included pre-season and in-season. The authors reported positive findings, BFlh fascicle length increased when compared to baseline (*d* = 1.99, p < 0.001), eccentric hamstring strength increased (*d* = 1.34, p = 0.026), maximal velocity increased by 3.4% (± 1.4%) at the end of pre-season, and horizontal force production increased by 9.7% in-season (± 2.2%). With the increasing popularity of hip-dominant FIT for hamstring specific adaptations, investigations into the effect of inertial load in such exercises is warranted. It is hypothesised that lower inertial loads will lead to higher peak power values, whereas higher inertial loads will lead to greater ECC overload values.

## MATERIALS AND METHODS

### Participants

Fourteen recreationally trained males (27.9 ± 6.4 years, 90 ± 10.7 kg, 180.7 ± 5.5 cm) participated in the study. All participants had a minimum of two years of resistance training experience including RDL training, although none had experience in FIT. Participants did not perform any strenuous exercise 48 hours before testing. Participants were medically screened and excluded if any musculoskeletal injuries had occurred six months before the intervention. All participants gave written informed consent before participation and were made aware of all experimental procedures and risks. Procedures followed the Declaration of Helsinki [WMA, 25] and its later amendments and were approved by the Research Ethics Committee of the Institute of Technology Carlow (code-C00232530).

### Procedure

All participants performed two familiarisation sessions using a similar protocol as that of the testing sessions. Two familiarisation sessions are recommended to find stabilisation in the values obtained in FIT [18]. Each participant attended two testing sessions. The first testing session and the last familiarisation session were separated by five days to avoid the effects of muscle fatigue and delayed onset of muscle soreness. Testing commenced with a dynamic warm-up that lasted approx. 15 mins. The warm-up consisted of five minutes of low-intensity jogging proceeded by five dynamic stretches, targeting the gluteal, hamstrings, adductors, quadriceps, gastrocnemius, and a set of 12 repetitions of RDL on the flywheel device at 0.050 kg·m². The testing session consisted of four sets of 12 repetitions of an RDL performed on a flywheel device (kBox 3, Exxentric, AB TM, Bromma, Sweden) and access effects of varying inertial loads on power variables. Both the first and second repetitions of each set were used to ‘increase momentum’ and were excluded from data analysis. It has been highlighted that the first repetitions of FIT are needed to get the flywheel up to the desired velocity and have been described as ‘waste repetitions’ and, if included, may skew findings [26]. Each set was performed using different inertial loads, these being 0.025, 0.050, 0.075, and 0.100 kg·m². The order of the inertial load setting was randomised for each participant. A five minute inter-set rest period was given to allow the cessation of any fatigue effects and enable adequate recovery.

Foot placement was standardised, with participants standing directly over the drive belt. To standardise the participants’ range of motion, a piece of tape was placed at mid-point between the most distal point of the tibial tuberosity and the talus’s most proximal point. This was the designated starting point of the RDL, and the endpoint was full hip extension. A strap is connected from the handle to the flywheel shaft through a hole in the platform. The exercise starts in the fully flexed position (i.e., bottom of the range of motion). During the CON phase of the RDL, the handle sets the flywheel in motion. During the ECC phase of the RDL, the flywheel continues this rotation and is slowed to a stop by ECC muscle actions resulting in a braking action. The next repetition is completed similarly, although the flywheel rotates in the opposite direction [27]. Participants were instructed not to shrug shoulders at full hip extension, and ankle extension was not allowed. The participants were instructed to perform the CON phase as fast as possible and resist the inertial force gently during the first third of the ECC action and then applying maximal effort to stop the movement at the end of the range of motion.

Verbal encouragement was given to the participants during both the familiarisation and testing sessions. During each repetition, both CON and ECC power was recorded employing a data reader and transmitter (Kmeter, Exxentric, AB TM, Bromma, Sweden) attached to the flywheel device. Data was then transmitted via Bluetooth to an iOS device (iPad mini, Apple Inc., Cupertino, CA, USA). This method of data collection has been used in previous research [28].

### Power Measurements

The variables used for data analysis were peak CON power, peak ECC power, and the ratio between ECC peak power and CON peak power, reported as the % ECC overload and calculated as (ECC peak power - CON peak power / concentric peak power) * 100.

### Statistical analysis

Data are presented as mean values ± standard deviation (SD). The Shapiro-Wilk test (S-W) was used to determine whether data were normally distributed. A one way repeated measure ANOVA was applied to investigate the effects of varying inertial loads on power variables. The Bonferroni test was used as a post-hoc test correcting for multiple comparisons. Effect sizes were calculated using Hedge’s g and can be interpreted as small, d = 0.2; medium, d = 0.5; and large, d = 0.8 [29]. Only moderate to large effects were reported. Significance was set at P ≤ .05 for all tests. All statistical analyses were carried out using the commercial software ‘Jeffrey’s Amazing Statistics Program’ (JASP version 09.1, University of Amsterdam, Netherlands).

## RESULTS

The Shapiro-Wilk test (S-W) determined that the data be normally distributed. The mean and standard deviation of different inertial loading effects on power variables are displayed in Table 1. For CON peak power, a significant difference (p < 0.001) was discovered between different inertial loadings (Table 1). Hedge’s g effect size showed a moderate effect between both 0.5 kg·m² and 0.100 kg·m² (0.71) and also 0.025 kg·m² and 0.75 kg·m² (0.78) while a large effect was discovered between 0.025 kg·m² and 0.1 kg·m² (0.95) inertial loadings. For ECC peak power, a significant difference (p < 0.001) was also discovered between different inertial loadings (Table 1). Hedge’s g effect size showed a moderate effect between 0.025 kg·m² and 0.1 kg·m² (0.68) and also 0.5 kg·m² and 0.1 kg·m² (0.65) inertial loadings. For ECC overload, a significant difference (p < 0.001) was discovered between different inertial loadings (Table 1). Hedge’s g effect size showed a moderate effect between 0.025 kg·m² and 0.1 kg·m² (0.55) inertial loadings.

**TABLE I:** Peak concentric power, peak eccentric power, and % eccentric overload during different inertial loads (mean ± SD)

	Inertial in kg·m^²^			
	0.025	0.050	0.075	0.100
**CON Peak (W)**	1120.35 ± 530.53 ^#$+^	947.75 ± 380.96 ^$+^	800.42 ± 201.23	728.08 ± 192.23
**ECC Peak (W)**	1050.13 ± 425.15 ^$+^	1033.59 ± 406.80 ^$+^	880.92 ± 227.84	817.13 ± 206.27
**% OL**	- 0.16 ± 31.55	9.82 ± 10.85 *	10.41 ± 12.66 *	13.8 ± 12.66 *

AVG = Average Power, CON Peak = Peak Concentric Power, ECC Peak = Peak Eccentric Power, % OL = % Eccentric Overload

* statistically greater than 0.025 kg·m^²^,

# statistically greater than 0.050 kg·m^²^,

$ statistically greater than 0.075 kg·m^²^,

+ statistically greater than 0.100 kg·m^²^.

## DISCUSSION

This study aimed to investigate the effect of four differential inertial loads (0.025, 0.050, 0.075, and 0.100 kg·m²) on both CON and ECC power output and ECC overload during the flywheel RDL. This findings suggest that the highest values for peak CON power were achieved at the lowest inertial load (0.025 kg·m²), while for peak ECC power, the highest values were achieved at low to medium loads (0.025 and 0.050 kg·m²). For ECC overload, no statistical difference (< 0.05) was found between medium to high loads (0.050, 0.075, 0.100 kg·m²), but it should be noted that the highest value was achieved at the highest inertial load (0.100 kg·m²).

The current study results yield similar findings to previous research [18, 19, 23] with the lowest inertial load showing the highest values (0.025 kg·m²). Specifically, 0.025 kg·m² showed the highest value (see Table 1) for both peak CON and ECC power. Although 0.05 kg·m² ECC power value may have been lower than 0.025 kg·m² it was statistically significantly higher than both 0.075 kg·m² (p < 0.001) and 0.100 kg·m² (p < 0.001) which again coincides with past research [18]. Generating high muscular power is strongly related to power output production [30] and associated with athletic success. With this in mind, practitioners need to be confident that the inertial load chosen will maximise power output and lead to warranted adaptions. Lower inertial loads may result in further neuromuscular adaptations (i.e. motor unit recruitment, firing frequencies), but this has yet to be proven [6].

Regarding maximal ECC overload, this current study results again correspond with those of Sabido et al. [18], who reported higher inertial loads led to higher ECC overload values. We found that the lightest load was the significantly lowest (0.025 kg·m²) value, and there was no statistically significant difference between medium to heavy inertial loadings (0.050, 0.075, 0.100 kg·m²). Precisely 0.100 kg·m² reported the highest value (see Table 1). Practitioners can be confident that higher inertial loads will lead to higher ECC overload. Contrary to our findings Martinez-Aranda and Fernandez-Gonzalo [19] reported no increase in ECC overload past 0.0375 kg·m², perhaps due to exercise choice. It is reasonable to suggest that a single joint leg extension exercise [19] recruits fewer muscles than a large muscle group exercise such as the flywheel RDL or bilateral quarter squat [18], and therefore the athletes may not have been able to fully resist and break inertial forces which lead to an ECC overload. Previous research suggests that the most effective technique to maximise ECC overload is gently resist the force during the first third of the ECC phase, then maximally decelerate the rotating flywheel and stop at the end range of motion [31]. It has been previously reported that larger inertial loads may increase the ECC phase’s length, which can modify the ECC overload stimulus [27]. Specific adaptations may include an increase in muscle cross-sectional area, force output, and fiber shortening velocities, all of which have the potential to benefit power production characteristics [27].

There are drawbacks to the current research. Because of the study’s limited sample size, the statistical power of the findings is relatively low. As a result, the study’s findings must be carefully interpreted.

## CONCLUSIONS

To the best of the author’s knowledge this is the first study to investigate the effects of inertial load in a flywheel hip extension exercise such as the flywheel RDL. Our findings suggest that a lower inertial load leads to higher peak CON and ECC power values, specifically 0.025 kg·m². Regarding ECC overload, then medium to higher loads (0.050, 0.075, and 0.100 kg·m²) may lead to higher values, with 0.100 kg·m² displaying the largest output. This information may guide intensity prescription when incorporating flywheel RDL into a training program.
